# An exploration on the machine-learning-based stroke prediction model

**DOI:** 10.3389/fneur.2024.1372431

**Published:** 2024-04-29

**Authors:** Shenshen Zhi, Xiefei Hu, Yan Ding, Huajian Chen, Xun Li, Yang Tao, Wei Li

**Affiliations:** ^1^Department of Blood Transfusion, Chongqing University Central Hospital, School of Medicine, Chongqing University, Chongqing, China; ^2^Medicine School of Chongqing University, Chongqing, China; ^3^Clinical Laboratory, Chongqing Emergency Medical Center, Chongqing University Central Hospital, School of Medicine, Chongqing University, Chongqing, China; ^4^Intensive Care Unit, Chongqing Emergency Medical Center, Chongqing University Central Hospital, School of Medicine, Chongqing University, Chongqing, China

**Keywords:** stroke, cytokines, machine learning, random forest model, prediction model

## Abstract

**Introduction:**

With the rapid development of artificial intelligence technology, machine learning algorithms have been widely applied at various stages of stroke diagnosis, treatment, and prognosis, demonstrating significant potential. A correlation between stroke and cytokine levels in the human body has recently been reported. Our study aimed to establish machine-learning models based on cytokine features to enhance the decision-making capabilities of clinical physicians.

**Methods:**

This study recruited 2346 stroke patients and 2128 healthy control subjects from Chongqing University Central Hospital. A predictive model was established through clinical experiments and collection of clinical laboratory tests and demographic variables at admission. Three classification algorithms, namely Random Forest, Gradient Boosting, and Support Vector Machine, were employed. The models were evaluated using methods such as ROC curves, AUC values, and calibration curves.

**Results:**

Through univariate feature selection, we selected 14 features and constructed three machine-learning models: Support Vector Machine (SVM), Random Forest (RF), and Gradient Boosting Machine (GBM). Our results indicated that in the training set, the RF model outperformed the GBM and SVM models in terms of both the AUC value and sensitivity. We ranked the features using the RF algorithm, and the results showed that IL-6, IL-5, IL-10, and IL-2 had high importance scores and ranked at the top. In the test set, the stroke model demonstrated a good generalization ability, as evidenced by the ROC curve, confusion matrix, and calibration curve, confirming its reliability as a predictive model for stroke.

**Discussion:**

We focused on utilizing cytokines as features to establish stroke prediction models. Analyses of the ROC curve, confusion matrix, and calibration curve of the test set demonstrated that our models exhibited a strong generalization ability, which could be applied in stroke prediction.

## 1 Introduction

Stroke is a common and severe clinical disease with a high incidence among the elderly (1). Stroke is classified into two main types: ischemic and hemorrhagic. Ischemic stroke is predominantly caused by cerebral vascular obstruction with atherosclerosis as its pathological basis, leading to local cerebral tissue ischemia and hypoxic necrosis. Ischemic stroke is a disease with high mortality, disability, and recurrence rates, which can result in irreversible damage to the central nervous system (2) in contrast, hemorrhagic stroke has a lower incidence and its pathological basis is mainly brain vascular injury and hypertension (3). Studies have demonstrated that cytokines are key factors in stroke development. During the course of stroke, certain cytokines are released into brain tissue, leading to neuronal damage and neuroinflammatory responses (4, 5). In the case of ischemic stroke, a large number of cytokines, such as tumor necrosis factor-alpha (TNF-α) and interleukin-1 (IL-1), are released by cells surrounding the ischemic area. To some extent, these cytokines exacerbate the severity of ischemic neuronal injury (6, 7). Furthermore, cytokines may also promote neuronal apoptosis and glial cell proliferation. In the event of cerebral hemorrhage, red blood cell breakdown products activate the immune cells, resulting in the release of various cytokines. These cytokines may also contribute to the disruption of the blood–brain barrier and the deposition of collagen in tissues, further intensifying the extent and scope of bleeding and ultimately leading to neurofunctional impairments (8, 9). Studies have shown that there is a high correlation between the concentration of cytokines in the blood of stroke patients and the prognosis and clinical manifestations of stroke. Elevated levels of cytokines, including IL-1β, TNF-α, and IL-6, in the bloodstream of stroke patients imply a higher likelihood of deterioration of their condition (10). These findings suggest a significant correlation between cytokine levels and stroke risk. Thus, the development of a stroke prediction model based on cytokines holds promise for improving disease prognosis.

Machine learning is one of the main tools in data mining and its application in the field of medicine is rapidly expanding. Specifically, in the area of stroke (11), commercially available machine learning algorithms have been widely applied in clinical practice. They can establish risk models by learning from existing medical test or survey data of patients. These models are used for disease prediction (12, 13), diagnosis of disease severity (14), and evaluation of disease prognosis (15, 16). Our study explored the value of machine-learning methods for stroke prediction. We collected clinical factors such as age, sex, and cytokine levels from 2,346 stroke patients to build three machine learning models: Support Vector Machine (SVM), Random Forest (RF), and Gradient Boosting Machine (GBM). The predictive performance of these models was compared and evaluated. Based on this exploration, this study provides new methods and tools for stroke diagnosis and prediction. By combining machine learning algorithms with cytokine features, it can improve the accuracy of stroke diagnosis and predictive capabilities for stroke patients, providing decision support for clinical physicians. This is of great significance for timely intervention and treatment of stroke patients, helping to improve patient treatment outcomes and prognosis.

## 2 Materials and methods

### 2.1 General clinical data

The study recruited a total of 2,346 stroke patients from the Chongqing University Central Hospital between July 16, 2020, and July 7, 2021. The inclusion criteria were as follows: A. All patients were diagnosed according to the criteria established by the Fourth National Conference on Cerebrovascular Diseases; B. Patients had not received any anticoagulation or antiplatelet therapy or related treatments within the past 2 weeks; C. Patients were 18 years of age or older; D. Patients or their legal guardians had signed informed consent forms. The exclusion criteria were: A. history of cerebral infarction caused by fat embolism or tumors; B. Presence of renal or hepatic dysfunction or arterial inflammation; C. Pre-existing heart disease.

Clinical laboratory tests and demographic variables at admission were collected, including age, gender, IL-2, IL-6, IL-1β, IL-8, TNF-α, IL-5, IFN-α, IFN-γ, IL-17, IL-12P70, IL-4, and IL-10.

### 2.2 Healthy control subjects

From the health examination department of the same hospital, 2,128 healthy control subjects were selected and matched in terms of sex and age, with the following inclusion criteria: A. with regular annual check-ups; B. Without history of atherosclerosis or other cardiovascular or cerebrovascular diseases; C. Without other severe systemic diseases, such as endocrine disorders, autoimmune diseases, blood disorders, or cancer.

### 2.3 Establishment of the prediction models

We randomly divided the enrolled patients into two groups: a training set (70%) and a validation set (30%). Subsequently, we organized the collected data. In the case of missing values, the MICE package was used for the imputation. Our data were then normalized and features with excessively low variance, low correlation with the labels, or high intercorrelations were removed. In the training set, we conducted 10-fold cross-validation with a total of 10 iterations, harnessing the grid search to select optimal parameter combinations for the three classification algorithms: RF: the Random Forest, GBM: the Gradient Boosting, and SVM: the Support Vector Machine. After training and modeling the data using these three algorithms, we obtained the prediction results from different models. The average accuracy over multiple iterations was computed to derive the final model score, along with the generation of the final model itself.

### 2.4 Evaluation of the prediction models

ROC curves were plotted for each model’s training and testing results and the Area Under the Curve (AUC) was calculated. By comparing the AUC values, we assessed the optimal model and further analyzed the contribution of each feature to the model. Using calibration (calibration curve and Brier score), the accuracy and reliability of the predictive model were comprehensively evaluated. Assessing the accuracy and precision of the model using a confusion matrix.

### 2.5 Statistical analysis

We conducted a statistical analysis of the collected data using the R package (V4.2.2). The distribution of quantitative data was evaluated using tests for homogeneity of variance and normality. Normally distributed quantitative data are represented as mean ± standard deviation, and group differences were examined using *t*-tests. Count data are expressed as percentages (%), and group differences were assessed using the chi-square test. Statistical significance was set at *p* < 0.05.

## 3 Results

### 3.1 Comparison on the clinical features

A total of 2,346 stroke patients participated in this study, with males accounting for 66% of the total, while females represented only 34% (Table 1). Their ages were generally older, primarily ranging from 50 to 70 years. Additionally, our study included a group of 2,128 healthy individuals (653 females and 1,475 males). In the analysis of the correlation between cytokines and stroke, it was found that the levels of IL-1β, IL-5, and IL-4 were significantly higher in stroke patients than in the normal group (*p* < 0.05), whereas the differences in IL-2, IL-8, TNF-α, IL-6, IFN-α, IFN-γ, IL-17, IL-12P70, and IL-10 between the two groups were relatively small (*p* > 0.05).

**Table 1:** Comparison on the clinical features.

Characteristic	Non-stroke, *N* = 2,128	Stroke, *N* = 2,346	Test statistic	*p*-value^1^
Sex, n (%)			4.43	0.035
F	653 (31%)	789 (34%)		
M	1,475 (69%)	1,557 (66%)		
Age, Median (IQR)	57 (47, 68)	58 (50, 70)	−1.47	0.14
IL-2, Median (IQR)	1.05 (0.52, 1.76)	1.09 (0.56, 1.94)	−0.91	0.4
IL-6, Median (IQR)	11 (4, 37)	13 (5, 39)	1.94	0.052
IL-1β, Median (IQR)	0 (0, 3)	0 (0, 3)	−2.32	0.020
IL-8, Median (IQR)	1 (0, 9)	2 (0, 9)	1.16	0.2
TNF-α, Median (IQR)	0.79 (0.03, 2.04)	0.81 (0.02, 2.11)	0.58	0.6
IL-5, Median (IQR)	2.15 (1.11, 3.45)	2.16 (1.01, 3.70)	−2.14	0.032
IFN-α, Median (IQR)	0.98 (0.45, 1.88)	1.04 (0.41, 2.13)	−0.99	0.3
IFN-γ, Median (IQR)	2.6 (1.0, 5.4)	2.7 (0.9, 5.4)	−1.11	0.3
IL-17, Median (IQR)	2 (0, 5)	2 (0, 4)	1.04	0.3
IL-12P70, Median (IQR)	0.81 (0.38, 1.32)	0.80 (0.27, 1.28)	−1.20	0.2
IL-4, Median (IQR)	0.88 (0.58, 1.21)	0.88 (0.58, 1.23)	2.70	0.007
IL-10, Median (IQR)	1.5 (0.9, 2.9)	1.7 (1.0, 3.0)	1.59	0.11

^1^Pearson’s Chi-squared test; Welch Two Sample *t*-test.

### 3.2 Clinical feature correlations analysis

**Figure 1:**
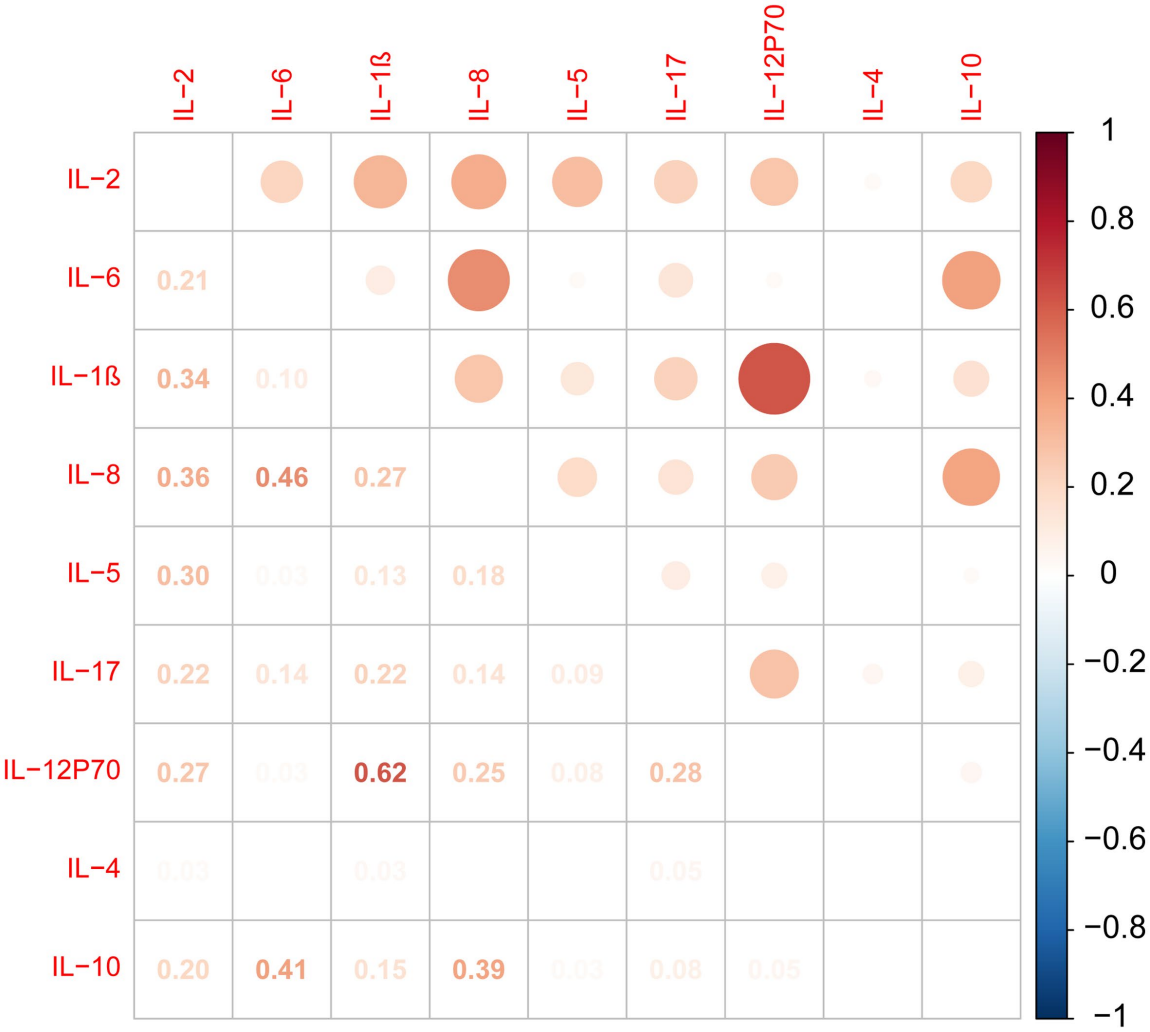
Cytokine correlations analysis.

According to the analysis of cytokine intercorrelations, our results indicate positive associations between IL-2 and IL-1β, IL-8, IL-5, and IL-12P70. IL-1β levels were positively correlated with IL-12P70, IL-8, and IL-17 levels. IL-6 levels were positively correlated with IL-8 and IL-10 levels. IL-8 levels were positively correlated with IL-5, IL-12P70, and IL-10 levels. IL-17 levels were positively correlated with IL-12P70 levels (Figure 1).

### 3.3 Establishment of the machine learning models

**Figure 2:**
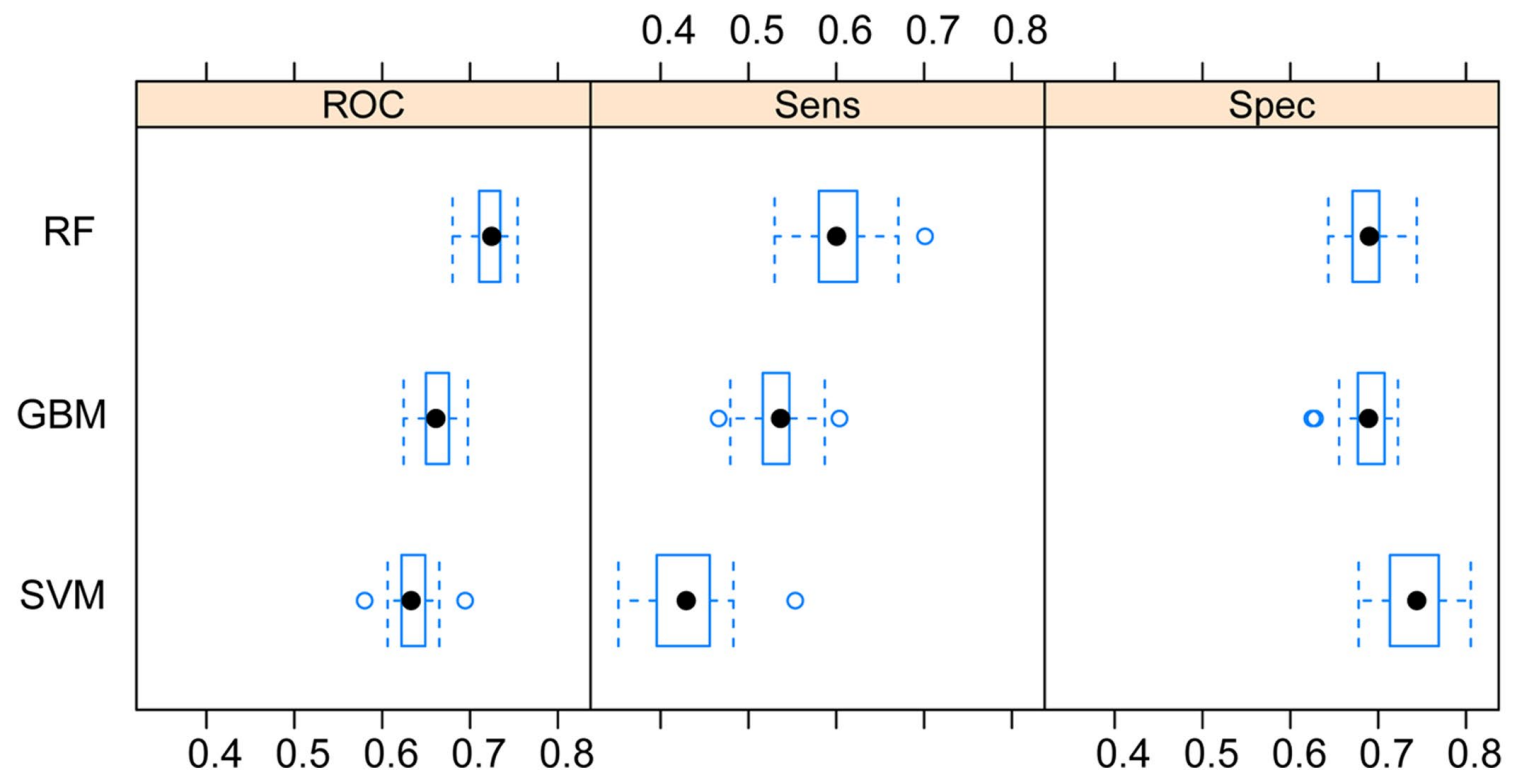
Comparison of the three machine learning models. RF, random forest; SVM, support vector machine; GBM, Gradient Boosting Machine.

After conducting 10 repetitions of 10-fold cross-validation and grid search, we trained our three machine learning models and evaluated their ROC area under the curve (AUROC), sensitivity (Sens), and specificity (Spec) on the training dataset. Box plots were generated for these metrics (Figure 2). Upon comparing these results, we observed that the RF model had the highest AUC value and sensitivity, with the smallest difference, and its specificity was only lower than the SVM model. Therefore, in this study, we chose the RF model as the final algorithm for establishing the model.

### 3.4 Predictive variable importance of RF model

**Figure 3:**
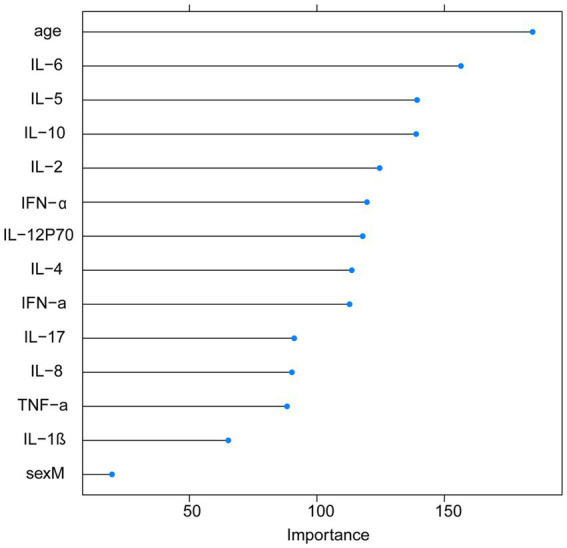
Feature importance rankings on account of the RF model.

With the visualization of the weight rankings, we showed a positive correlation between the importance of variables and the length of the corresponding bar in the bar graph. The research findings revealed that the top four features in terms of importance scores were age, IL-6, IL-5, and IL-10 (Figure 3).

### 3.5 The validation on models

**Figure 4:**
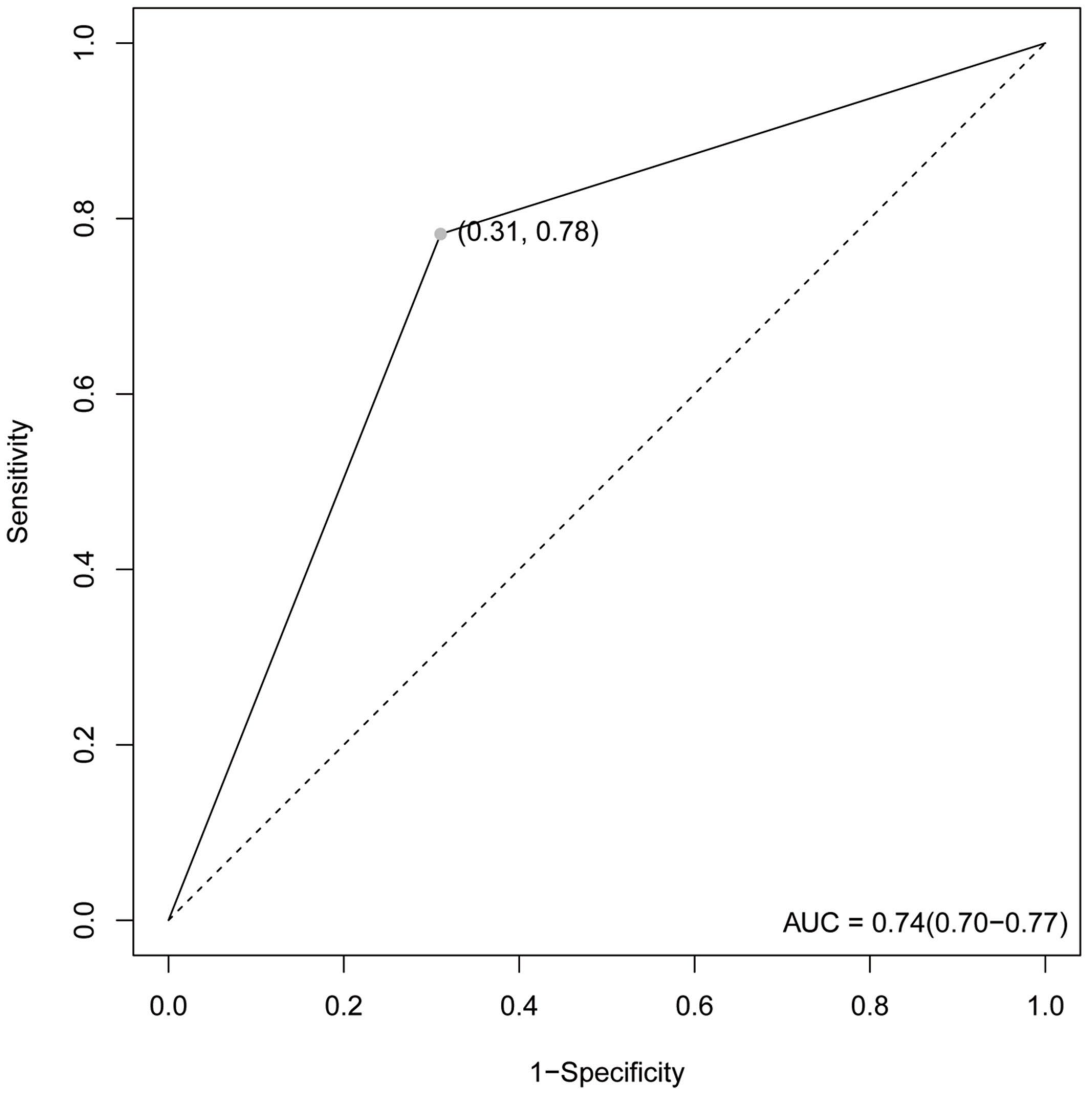
The ROC curve. The maximum value is 1. The point in the upper left corner is the optimal threshold; Specificity and Sensitivity are listed in parentheses.

**Figure 5:**
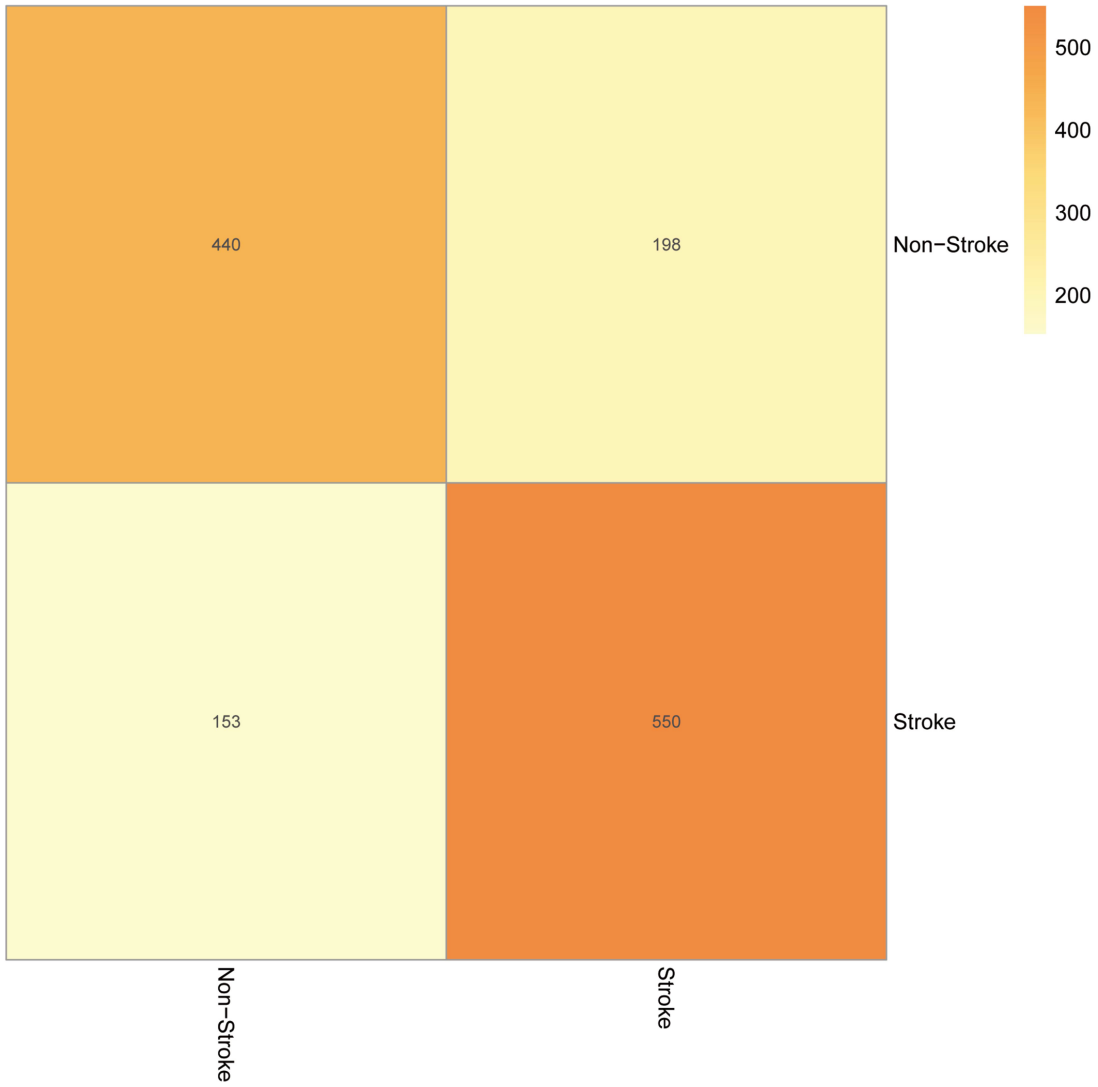
The confusion matrix.

Our test set was used to evaluate the trained model’s performance on new, unseen data, revealing an AUC value of 0.74 and a sensitivity and specificity of 0.31 and 0.78, respectively, (Figure 4). The prediction model was assessed using a test set. In classification problems, a confusion matrix is commonly used to measure the predictive performance of a classifier model. By comparing the classifier’s output with the actual labels, we obtained an accuracy of 0.738, a precision of 0.690, a recall of 0.742, and an F1-score of 0.715 (Figure 5).

**Figure 6:**
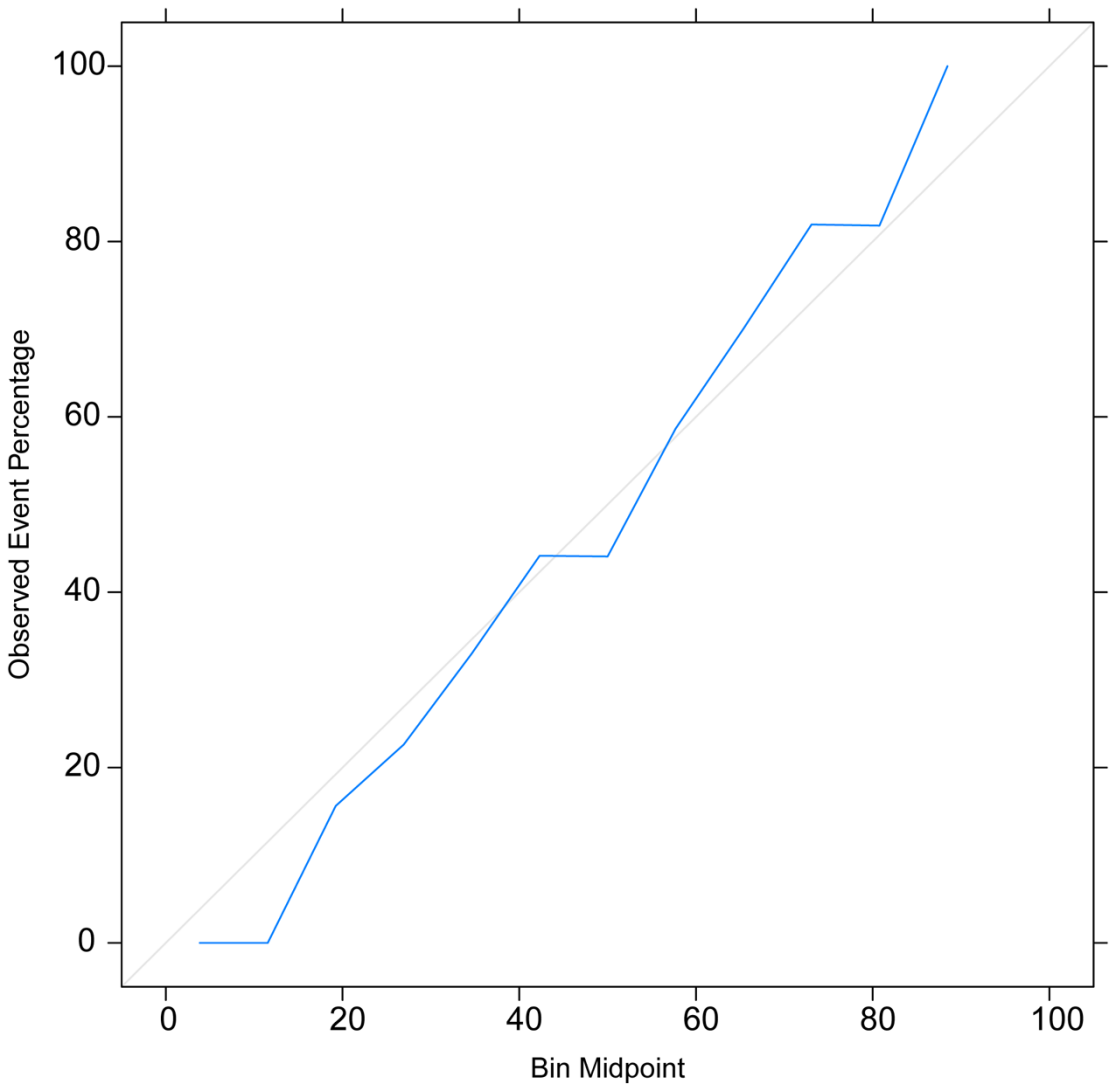
The calibration curve.

The calibration curve indicated that the prediction model exhibited small errors between the predicted and actual values, demonstrating its high accuracy (Figure 6). This further confirms the good generalization capability of the RF model.

## 4 Discussion

Stroke, also known as cerebrovascular accident, refers to a series of clinical syndromes caused by cerebrovascular diseases including localized cerebral ischemia or hemorrhage. It is characterized by rapid progression, often resulting in varying degrees of disability in patients, leading to a decreased quality of life and an increased burden on families and society (17). Therefore, early, timely, and effective treatment is crucial for saving lives and reducing the occurrence of severe sequelae.

In the prediction and diagnosis of stroke, relevant features can be extracted from a large amount of information, such as medical images or clinical data. Feature extraction is a key step in stroke machine-learning applications, as machine-learning algorithms are widely used for feature classification and prediction. By applying machine learning algorithms to stroke, we developed a novel approach to diagnosis and treatment that surpasses manual judgment in sensitivity and significantly improves the accuracy of stroke diagnosis and treatment (11). For instance, image processing techniques based on machine learning algorithms can assist clinicians in identifying specific subtypes of cerebral hemorrhage, thereby enhancing clinical outcomes (18). Furthermore, they can predict the severity and occurrence of stroke symptoms, providing crucial insights for making clinical decisions (19).

In this study, we employed three machine learning algorithms, SVM, RF, and GBM, and incorporated 14 features consisting of clinical laboratory indicators and demographic variables to establish our stroke prediction model. Our findings demonstrate that the RF model outperformed both the GBM and SVM models in terms of AUC values and sensitivity on the training dataset. Furthermore, by utilizing the RF algorithm to rank the importance of the features, it was observed that IL-6, IL-5, IL-10, and IL-2 obtained higher importance scores and were ranked among the top features.

One key influencing factor in stroke is a dysregulated inflammatory response, which could lead to neuronal damage, impaired neural function, and further injury after brain damage (20). Cytokines are a group of proteins that regulate immune and inflammatory responses and are closely associated with the occurrence of stroke. Following stroke, a significant amount of cytokines is produced, triggering an inflammatory response that results in neuronal cell death and exacerbation of brain damage. Cytokines released during stroke, such as TNF-α, IL-1β, and IL-6, further activate the inflammatory response and induce cell death (21). Other neurons distant from the injured area might also suffer indirect damage, which is mediated by cytokines and worsens stroke conditions.

IL-6, IL-5, IL-10, and IL-2 are important inflammatory mediators involved in the regulation of multiple biological processes, including immune modulation and inflammation, and are also associated with the pathogenesis of many diseases (22). Studies have shown that serum levels of IL-6, IL-5, IL-10, and IL-2 in stroke patients are closely correlated with stroke prognosis (23, 24). Experimental studies using mouse models have demonstrated the involvement of IL-6 in the process of neuronal redeath following stroke (25). After stroke, IL-6 is capable of modulating the function of stromal cells, promoting neuronal damage, and adversely affecting the recovery of neurological function (26). It has been revealed that IL-5 reduces the mortality rate of neurons after cerebral infarction, promotes neuronal regeneration and recovery, and regulates the post-stroke injury microenvironment. Following brain injury, TNF-α levels increase, which correlates with the severity of the injury. However, IL-10 inhibits TNF-α and decreases the inflammatory response after brain injury (27). Clinical experiments have demonstrated significantly lower levels of IL-2 in the serum of patients with acute stroke than in the normal control group (28). IL-2, by stimulating T cell activation and proliferation, regulates the immune response after stroke and influences the differentiation of TH1/TH2 cells and related factors, thereby modulating the immune response (29). These findings highlight the extensive biological effects of cytokines on the pathogenesis of stroke.

The AUC value, confusion matrix, and calibration curve are important indicators for measuring the predictive ability of classification models. The evaluation of predictive ability of the model in this study shows an AUC of 0.74, indicating a moderate level of predictive ability. The sensitivity is 0.31, and the specificity is 0.78. The low sensitivity implies a certain level of missed diagnosis in identifying stroke patients by the model, while the high specificity suggests good performance in excluding non-stroke patients. The confusion matrix results show that the accuracy, precision, recall, and F1 score are all around 0.7, indicating that the model has some predictive ability overall but there is room for improvement. The calibration curve demonstrates small errors between predicted and actual values, confirming the accuracy and generalization ability of the model. However, this study has limitations in terms of analysis. First, we acknowledge that the study has limitations in terms of data sources, as the training data originates from a single source and lacks diverse datasets from different demographic groups or geographical locations. This may result in insufficient generalizability of the model on other datasets, thus requiring further validation and expansion. Second, machine learning algorithms are often considered black box models, making it difficult to explain their predictive results. This is also an important issue in current machine learning research. To address this problem, we need to deepen our understanding of the internal workings of machine learning algorithms, as well as how to explain their results to clinical physicians and patients. Future research should continue to explore these issues and strive to improve the interpretability and generalizability of machine learning algorithms. In summary, while machine learning methods offer some improvements in stroke risk prediction, their actual significance in clinical settings requires further evaluation and validation. The model should be integrated as part of clinical decision support tools, combined with clinical judgment, to maximize the identification and management of stroke patients.

## 5 Conclusion

In this study, we focused on utilizing cytokines as features to establish stroke prediction models. Analyses of the ROC curve, confusion matrix, and calibration curve of the test set demonstrated that our models exhibited a strong generalization ability, which could be applied in stroke prediction.

## Data availability statement

The original contributions presented in the study are included in the article/Supplementary material, further inquiries can be directed to the corresponding author.

## Ethics statement

The studies involving humans were approved by the Ethics Committee of Chongqing Emergency Medical Center and Chongqing University Central Hospital (Approval Ethics Review No. 2021-74). The studies were conducted in accordance with the local legislation and institutional requirements. The participants provided their written informed consent to participate in this study.

## Author contributions

SZ: Writing – review & editing, Writing – original draft, Resources, Project administration, Methodology, Investigation, Formal analysis, Data curation, Conceptualization. XH: Writing – review & editing, Writing – original draft, Supervision, Investigation, Data curation. YD: Writing – review & editing, Writing – original draft, Methodology, Investigation. HC: Writing – review & editing, Writing – original draft, Methodology, Investigation. XL: Writing – review & editing, Supervision, Data curation. YT: Writing – review & editing, Data curation. WL: Writing – original draft, Resources, Project administration, Methodology, Funding acquisition, Formal analysis, Conceptualization.
